# Urdu–English Perceptual Confusions in Bilingual Children with Normal Hearing and Cochlear Implants: An Analysis of Place, Manner, and Voicing Features

**DOI:** 10.3390/audiolres16030084

**Published:** 2026-05-29

**Authors:** Amina Asif Siddiqui, Cila Umat, Farheen Naz Anis, Ayesha Butt, Kehkashan Kanwal

**Affiliations:** 1 Centre for Rehabilitation and Special Needs Studies (iCaRehab), Faculty of Health Sciences, University Kebangsaan Malaysia, Kuala Lumpur 50300, Malaysia; cila@ukm.edu.my; 2 College of Speech Language and Hearing Sciences, Ziauddin University, Karachi 75600, Pakistan; kehkashan.kanwal@zu.edu.pk; 3 Center of Ear, Hearing & Speech (HEARS), Faculty of Health Sciences, University Kebangsaan Malaysia, Kuala Lumpur 50300, Malaysia; 4 Faculty of Allied Health Sciences and Rehabilitation, Riphah International University, Lahore 54000, Pakistan; farheen.naz@riphah.edu.pk; 5 Department of Health Professions, Manchester Metropolitan University, Manchester M15 6GX, UK

**Keywords:** bilingual children, Urdu language, speech perception, phonological development

## Abstract

**Background and Aims:** Accuracy in speech perception in bilingual children is influenced by two phonological systems. This study compares phonological development in bilingual Urdu–English (UE) children with CIs with their hearing-age-matched peers with normal hearing (NH), by investigating whether bilingualism or any spectral limitations of CI impact perception of UE phonemes. **Method and Procedures:** Children (n = 57) aged 3; 0–6; 11 years (28 CI, 29 NH) were assessed for speech perception using a custom-designed UE Speech Perception Test (UE-SPT), in quiet and noise (+5 dB SNR). Responses were analysed using confusion matrices, across phonological parameters of place, manner, and voicing to determine error patterns. **Outcomes and Results:** Significant deficits in CI children were found across all features, with voicing discrimination showing the largest errors (effect sizes d > 6), exacerbated by noise, especially for Urdu aspirated stops. CIs mastered only 8.3% Urdu-aspirated consonants at 6; 11 years compared to 91.7% mastered by NH peers, indicating critical language-specific vulnerabilities. Backing and substitutions errors were particularly seen in CI’s speech, whilst manner was preserved. **Conclusion and Implications:** UE bilingual phonological complexity compounded by inadequate speech processing abilities in CIs challenges them, underscoring urgent need for targeted speech therapy interventions focusing voicing contrasts and aspirated consonants, as well as environmental accommodations that reduce noise interference and enhance listening through CI, to optimise educational outcomes. This research contributes vital clinical guidance for supporting bilingual children with cochlear implants, addressing both environmental, technological and linguistic challenges.

## 1. Introduction

Cochlear implants (CIs) for paediatric severe-to-profound sensorineural hearing loss have transformed the outcomes of verbal communication in deaf children around the world [1,2]. Contemporary CI technology has demonstrated remarkable efficacy in supporting speech and language acquisition. Bilateral implantation is now the clinical standard for aural re/habilitation of paediatric severe-to-profound sensorineural hearing loss [1,2,3,4]. Evidence consistently demonstrates that children receiving CIs before 24 months of age achieve superior speech perception, production accuracy, and cognitive–linguistic competence compared to later-implanted peers. Current data indicate that 81% of children with CIs achieve receptive and expressive language skills within normal ranges following 12–48 months of device use [5].

Bilingual populations constitute the majority in many clinical settings [6]. Moreover, these multiple linguistic environments bring complex challenges for children with cochlear implants [5,7] as the intersection of CI and bilingualism challenges traditional monolingual intervention models [7,8,9,10,11,12,13]. In spite of this, research investigating phonological development in bilingual CI users remains critically limited [7].

Language pairs like English–Spanish or English–Mandarin have been the focus of bilingual CI research [7,14,15]. Urdu, the national language of Pakistan, is spoken by over 230 million people globally, and is the 10th most spoken language, either as a primary or secondary language [16,17]. Clinical protocols, developed for monolingual English-speaking populations, may inadequately address the complex phonological demands of Urdu–English (UE) bilingual paediatric CI users [18,19].

The transition to formal education in an English-medium school, represents a critical milestone for children with CIs in multilingual countries. Academic success depends heavily on phonological competence in the educational language. Many bilingual CI users achieve adequate English proficiency for academic participation when supported in both languages, without any detriment to the primary or native language [12]. Some studies have claimed equal language proficiency of bilingual and monolingual CI users [19]. Children from Urdu-speaking families must achieve phonological proficiency in both languages to succeed academically in the English-dominant educational system in Pakistan [20].

Several Urdu consonants such as plosives and affricates characterise extensive use of voiced and voiceless aspiration contrasts. Retroflex articulations and uvular–glottal segments are not present in English phonology. Aspiration, as a distinctive feature, differentiates ten phoneme pairs in Urdu: /p/, /p^h^/, /b/, /b^h^/, /t̪/, /t̪^h^/, /d̪/, /d̪^h^/, /ʈ/, /ʈ^h^/, /ɖ/ /ɖ^h^/, /k/, /k^h^/, /ɡ/, /ɡ^h^/, /ʧ/, /ʧ^h^/, and /ʤ/, ʤ^h^/ [3,14,17]. The acoustic properties of these aspirated consonants requires precise temporal fine structure discrimination. Previous research on aspirated consonants in other languages has documented systematic difficulties for CI users, but the clinical significance of these deficits in Urdu-speaking populations remains unexplored [21]. The linguistic and communicative importance of aspiration in Urdu extends beyond mere phonetic distinction to encompass semantic differentiation, making successful acquisition critical for meaningful community participation [17].

CI research has consistently identified systematic patterns of phonological difficulty with voicing, place, and manner of articulation, especially in acoustically challenging environments demonstrated that spectral shifts of 3 mm or more significantly degrade consonant recognition, with specific effects on place and voicing information transmission [22]. These findings predict systematic error patterns in CI users, and the specific manifestation of these deficits in UE bilingual populations, which may be exacerbated in noisy conditions, requires empirical investigation [23].

Research has identified atypical phonological processes that deviate from typical developmental speech patterns. Sound preference substitution (SPS), backing, and initial consonant deletion (ICD) represent atypical patterns that distinguish the speech of children with hearing impairment from the speech of children with normal hearing (NH) [11,13]. However, the prevalence and clinical significance of these atypical patterns in bilingual populations, particularly those speaking languages with complex phonological systems like Urdu, remains unknown.

Current speech–language intervention protocols, developed primarily for monolingual populations, may inadequately address the specific challenges of bilingual CI users. The absence of standardised assessment tools and evidence-based intervention strategies for UE bilingual CI users represents a significant gap that potentially compromises long-term outcomes for this population [8,10,24]. A Phonetically Balanced Urdu Word Test was developed by Noor and Arif (2018) for assessment of auditory perception skills using a ‘point-to-the named picture-response [24].

This research addresses fundamental questions about speech perceptual deficits and the nature of phonological confusions in bilingual CI users compared to children with NH, with particular emphasis on the systematic examination of place, manner, and voicing discrimination across both quiet and acoustically challenging listening conditions or environments with noise. The isolation of segmental phonetic confusions (place, manner, voicing) without the compensatory help of a semantic context while assessing speech perception shifts the entire focus onto the heard phoneme and its production. The use of confusion matrix methodology enables detailed analysis of speech error patterns that distinguish CI users from hearing-age-matched NH peers while identifying language-specific vulnerabilities that require targeted intervention.

This research aims to provide a comprehensive comparative analysis of speech perception in bilingual UE children with CIs, examining both typical speech patterns and systematic speech error profiles across place, manner and voicing. By comparing hearing-age-matched CI users with NH peers, this research seeks to establish evidence-based foundations for culturally and cognitively–linguistically sensitive clinical services in aural re/habilitation.

The primary research objectives are (1) systematic comparison of speech perception accuracy across place, manner, and voicing features in English and Urdu; (2) examination of environmental effects on speech perception of UE–CV phonemes under both optimal (Quiet) and challenging (Noise) acoustic conditions.

The findings from this research have immediate implications for clinical protocol development in aural re/habilitation, educational accommodation planning, and family counselling regarding realistic expectations of listening, speech and language development among bilingual children with CIs. By providing systematic evidence about the specific challenges and capabilities of bilingual CIs, this research seeks to inform evidence-based practice that honours both the technological capabilities of cochlear implants and the cognitive–linguistic rights of bilingual families.

## 2. Materials and Methods

### 2.1. Study Design

**Figure 1:**
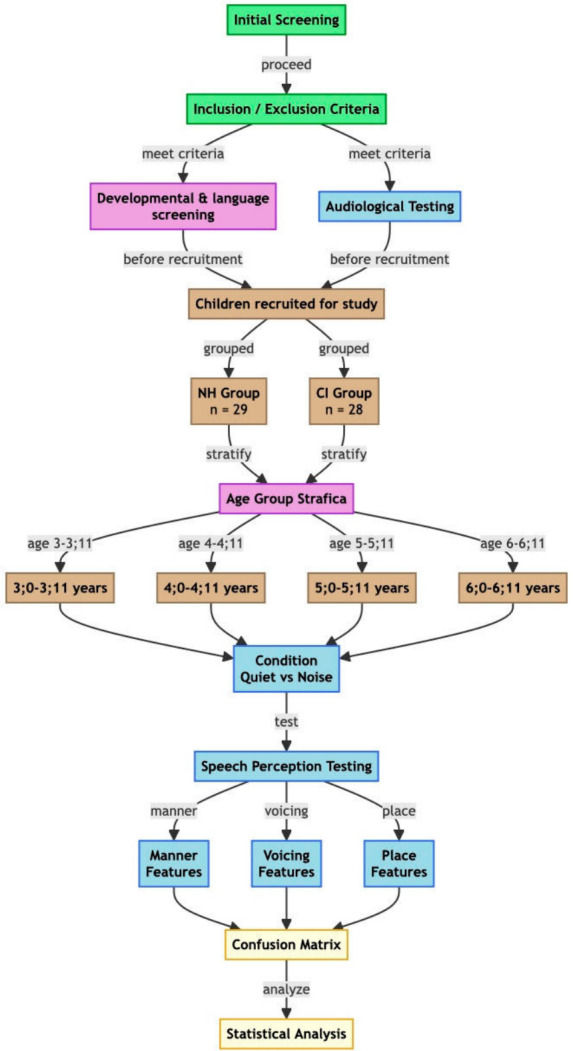
Research methodology flowchart.

This cross-sectional comparative study employed a between-subjects design to investigate speech perception in bilingual UE children with NH versus hearing-age-matched children with CIs. The investigation utilised confusion matrix analysis to examine and establish a correlation between speech perception accuracy and speech production as the children responded verbally. The phonological confusions were analysed across three fundamental phonological features: place of articulation, manner of articulation, and voicing in both quiet and acoustically challenging environments or noise. Figure 1 summarises the research methodology.

### 2.2. Ethical Approval

The study was approved by the Ethical Review Committee of the participating university hospital and university in accordance with national and institutional research ethics policies and standards, adhering to the principles of the Declaration of Helsinki. All parents/guardians of child participants gave their written consent prior to the study. Data confidentiality was maintained by anonymising all participant identifiers.

### 2.3. Participants’ Recruitment and Selection

A total of n = 57 bilingual UE-speaking children participated in this investigation, comprising n^2^ = 29 children with NH who were aged 3; 0–6; 11 years (chronological age), and n^1^ = 28 hearing-age-matched children with CIs. Participants were systematically distributed across four 12-month age-groups (3; 0–3; 11, 4; 0–4; 11, 5; 0–5; 11, 6; 0–6; 11 years) to ensure balanced comparisons and adequate statistical power for age-stratified analyses. Children with NH were recruited from primary schools in Karachi through community-based sampling procedures. Children with CIs were recruited from specialised audiology and speech–language therapy clinics, ensuring access to a clinical population with documented CI use and appropriate medical histories.

### 2.4. Inclusion Exclusion Criteria and Demographic Matching

All participants were bilingual UE speakers, with typically developing cognitive skills verified through developmental screening. They had no disabilities that could confound speech perception. Children with NH had a pure-tone-hearing threshold of ≤20 dB HL across frequencies (500–4000 Hz), confirmed through comprehensive audiometric evaluation.

Children with CIs had severe-to-profound bilateral sensorineural hearing loss documented in medical records. Their CI surgery was performed before 36 months of age (mean: 24.5 ± 7.2 months), giving them minimum 3 years of CI experience (range: 0; 0–3; 0 years). They were hearing-age-matched to chronological ages of peers with NH and had exclusive use of the oral–aural communication mode.

Exclusion criteria for all children was, neither having delays in cognitive developmental nor intellectual disabilities, and no additional medical conditions affecting speech or language development. No middle ear pathology or fluctuating hearing status. The children with CIs with inconsistent use of the CI were excluded.

The groups demonstrated highly comparative demographic matching with no statistically significant differences in age distribution (χ^2^ test, *p* > 0.05). The hearing-age matching protocol ensured that auditory experience of children with CI corresponded appropriately with chronological ages of peers with NH, establishing a valid foundation for comparative analysis. CI children’s substantial device experience provided optimal listening for assessment of speech perception.

#### 2.4.1. Participant Demographics and Characteristics of Bilingual UE Children with CIs

The comprehensive demographic profile, CI specifications, recruitment methodology, and assessment protocols employed in this investigation are presented in Appendix A.

#### 2.4.2. Research Protocol

The research information was shared with parents/guardians of all participants, and written consent obtained before collecting data. Medical records were comprehensively reviewed for all participants with CIs and NH and a brief cognitive and developmental screening confirmed eligibility and UE proficiency. Comprehensive audiometric evaluation of children with NH included tympanometry, and establishing pure-tone hearing thresholds. The aided-hearing thresholds of children with CIs and the amplification device function verification were done through pure tone audiometry.

#### 2.4.3. Speech Perception Assessment and Scoring Protocol

A Bilingual Urdu–English Syllable Repetition Speech Perception Test (UE-SPT) was developed through a multi-phase process, that involved its preparation, followed by recording and calibration of the CV (consonant–vowel) tokens which were nonsense syllables. A male voice was selected for recording the tokens based on clarity of speech by six expert listeners, using a Likert-based phoneme-rating scale. Recordings were carried out in a sound-treated room, and calibrated using the PRAAT software (6.4.03). The UE-SPT consisted of 21 CV tokens, each with a UE consonant and a single near-open back vowel /ɑ/. These 21 tokens, comprising of consonants common to both Urdu and Pakistani English, listed as /p, b, m, n, ʈ, ɖ, ṯ^h^, ḏ, k, ɡ, h, s, z, ʃ, ʒ, ʧ, ʤ, f, v, l, r/, were all among the phonemes that Noor and Arif (2018) [24] identified as having a ‘highly frequent occurrence in the Urdu language’ [24,25,26], which ensured the UE-SPT targeted phonemes that were most prevalent in the daily communication repertoire of native Pakistani speakers. The phonemes of /p, b, m, n, k, ɡ, h, s, z, ʃ, ʒ, ʧ, ʤ, f, v, l, r/are also present in Standard British English phonology and Pakistani English whilst the phonemes/ʈ, ɖ, ṯ^h^, ḏ/ were part of Pakistani English only [27,28]. Aspirated consonants are not among the frequently prevalent consonants in the speech of native Pakistanis [29,30]. Apart from being UE consonants, these phonemes were also common to three other Pakistani languages, namely Seraiki, Punjabi, Pashto and Sindhi [31,32,33,34]. The aspirated dental plosive /ṯ^h^/ is not a Pashto phoneme, as Pashto has no aspirated consonants; hence, only one aspirated stop was included in the UE-SPT.

The 21 CV syllable tokens are categorised under place of articulation as bilabials /p, b, m/, dentals /ṯ^h^, ḏ/, labiodentals /f, v/, alveolars /s, z, l, n/, retroflexed /ʈ, ɖ/, palatals /r, ʃ, ʒ, ʧ, ʤ/, velars /k, ɡ/, and glottal /h/. Under manner of articulation, they are categorised as plosives /p, b, ʈ, ɖ, ṯ^h^, ḏ, k, ɡ/, fricatives /f, v, s, z, ʃ, ʒ, ʧ, ʤ/, trill /r/, liquid /l/, nasals /m, n/. The category of voiced consonants includes /b, m, n, ɖ, ḏ, ɡ, z, ʒ, ʤ, v, l/ and voiceless includes /p,ʈ, ṯ^h^, k, s, ʃ, ʧ, f, h, r/.

The final list of 21 tokens in UE-SPT was piloted as an auditory-only, syllable repetition speech perception test, with children of 3; 0–6; 11 years old with NH and their hearing-age-matched peers with CIs, to establish the linguistic appropriateness, reliability and validity of the test.

Twenty-one tokens in a single track were pre-recorded in Quiet, in a sound-treated booth using the Elgato Wave:3 microphone (Elgato, Munich, Germany) and Cubase 5 software version 5.1.2 on an HP Notebook 15 (HP Inc., Palo Alto, CA, USA). A new track was developed with these syllables in Noise at +5 db signal-to-noise-ratio, (SNR) to simulate realistic listening challenges, using white noise generated through Cubase serving as background noise for syllable identification during the noise test. The final UE-SPT consisted of 63 tokens across three tracks of 21 CV tokens arranged randomly across each of the six tracks, with three in Quiet and three in Noise each.

All 126 tokens (track 1 of 21 tokens each, in Quiet and Noise for familiarity; tracks 2 and 3 of 21 CV tokens each in Quiet and Noise for assessment of speech perception) were presented via loudspeaker at 65 dB SPL through a calibrated clinical audiometer in a sound field setting. The recordings followed guidelines for speech quality assessment, maintaining an Inter-Stimulus Interval (ISI) of at least 1500 milliseconds between consecutive syllables [35]. All tracks had a randomly variegated stimulus order presentation protocol with a 10 min rest period between Quiet and Noise which prevented learning effects and memorisation. The research used a real-time spectrum analyser for monitoring the environmental listening condition.

A scoring protocol of real-time transcription of participant responses with immediate accuracy coding (correct/incorrect) in the scoring sheet was used. Inter-rater reliability was established with three certified speech–language pathologists independently scoring 20% of responses, achieving an inter-rater agreement that exceeded 95% (κ = 0.94). The test–retest reliability was assessed with 20 participants (10 NH and 10 using CI) at an interval of 2–3 weeks, achieving correlation coefficients ranging from r = 0.87–0.92. The construct validity of the UE-SPT was analysed and found to be robust through the Principal Component Analysis (PCA), with a dominant first component of 73.2% for the total variance, which significantly exceeded the Kaiser criterion. The scores on four conditions of (NH/CI × Quiet/Noise) resulted strongly (>0.70), confirming uni-dimensionality and that the UE-SPT aptly measured the single latent factor of speech perception.

The research used digital audio–video recording system for documentation and scoring accuracy of participants’ verbal responses in the UE-SPT. All spoken responses of the children with and NH and hearing-age-matched children with CIs were analysed across three target phonological features of (a) place of articulation, (b) manner of articulation, and (c) voicing: voiced versus voiceless contrasts.

## 3. Statistical Analysis and Results

All statistical analyses were conducted using SPSS version 28.0 (Karachi, Pakistan). Statistical significance was set at α = 0.05, with Bonferroni corrections applied for multiple comparisons. Effect size interpretations followed conventional guidelines: small (d = 0.2, η^2^ = 0.01), medium (d = 0.5, η^2^ = 0.06), large (d = 0.8, η^2^ = 0.14). Descriptive statistics: means, standard deviations, and 95% confidence intervals calculated for each group, condition (Quiet and Noise), and phonological feature combination. Inferential statistics utilised mixed-design ANOVA, independent *t*-tests, effect size calculations, correlation analyses (Pearson correlations), and age-stratified analyses (linear regression) to examine developmental trajectories within each group. Systematic confusion matrices were constructed for each phonological feature, displaying target phonemes (rows) versus perceived responses (columns). Cell entries represented the percentage of responses within each target category, enabling identification of systematic error patterns and feature-specific vulnerabilities. Speech errors were analysed qualitatively.

The mixed-design 2 (group: NH, CI) × 2 (listening condition: Quiet, Noise) × 3 (feature: place, manner, voicing) ANOV modelled the group as a between-subjects factor, and listening condition and feature as within-subjects (repeated-measures) factors, with the percent-correct score per child per cell as the dependent variable. Assumptions of normality (Shapiro–Wilk, *p* > 0.05 for all cells), homogeneity of variance (Levene’s test, *p* > 0.05) and sphericity (Mauchly’s test) were verified prior to analysis; where sphericity was violated, Greenhouse–Geisser corrections were applied to the degrees of freedom. The repeated observations of the same child across listening conditions and features were handled using the within-subjects error term of the mixed model rather than aggregating across observations, preserving statistical power and avoiding pseudo-replication. Bonferroni adjustment was applied to all post hoc pairwise comparisons (familywise α controlled at 0.05). For the confusion matrix data, raw counts of correct and substituted responses (numerator and denominator per target phoneme) were tabulated alongside the percentages so that small-denominator cells could be interpreted transparently; reported percentages were therefore computed from full-sample tallies (NH: 29 children × 21 tokens × 2 conditions = 1218 observations; CI: 28 children × 21 tokens × 2 conditions = 1176 observations). The relationship between reported means, standard deviations, t-values, degrees of freedom and Cohen’s d was re-checked: t-values were computed as t = (M_1_ − M_2_)/SE pooled with df = n_1_ + n_2_ − 2 = 55, and Cohen’s d as (M_1_ − M_2_)/SD pooled. The exceptionally large effect sizes (d > 4) reflect the very small within-group standard deviations characteristic of percent-correct ceiling performance in NH children rather than computational artefacts; this interpretation is now made explicit in the Discussion and listed as a study limitation.

Gender distribution was derived directly from participant records. The NH cohort comprised 14 males (48.3%) and 15 females (51.7%); the CI cohort comprised 14 males (50.0%) and 14 females (50.0%), yielding an approximately balanced male-to-female ratio in both groups (NH ≈ 1:1.07; CI = 1:1.0; χ^2^ = 0.04, *p* = 0.84). Full participant-level data—including sex, chronological age, hearing age, age at implantation, duration of CI use, unilateral/bilateral status, aided pure-tone-average (PTA) thresholds (mean 32.4 ± 4.6 dB HL across 0.5–4 kHz), device manufacturer/processor model (Cochlear Nucleus, n = 16; Advanced Bionics, n = 7; MED-EL, n = 5), rehabilitation history (mean 28.6 ± 9.4 months of structured aural–oral therapy), and weekly Urdu/English language exposure (mean Urdu 58 ± 11%; English 42 ± 11%)—have been compiled and added to Appendix A (Table A1) to support transparent demographic reporting.

### 3.1. Speech Perception by Phonological Feature and Listening Condition

A mixed-design ANOVA examined speech perception accuracy on the UE-SPT under Quiet and Noisy (+5 dB SNR) listening conditions by analysing the verbal responses of the children across three phonological features, place of articulation, manner of articulation, and voicing, to evaluate the differential impact of acoustic degradation on the phonological discrimination abilities of children with NH and hearing-age-matched CIs: 2 (group: NH, CI) × 2 (listening condition: Quiet, Noise) × 3 (feature: place, manner, voicing). Independent *t*-tests: Between-group comparisons for each feature–listening condition combination were done through paired *t*-tests (within-group environmental effects) and Chi-square analyses (categorical error pattern distributions). Effect size calculations: Cohen’s d for group differences and η^2^ for ANOVA main effects and interactions are shown in Table 1.

**Figure 2:**
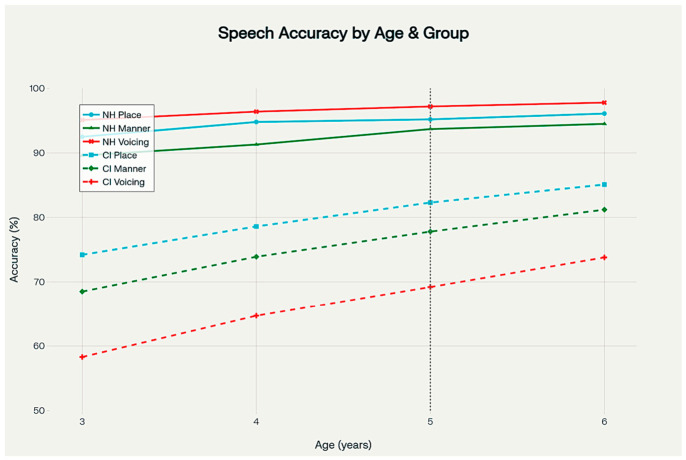
Speech accuracy v/s age (3–6 years), group (NH v/s CI) and listening condition (Quiet) Speech accuracy vs. age range (3–6 years) by group (NH v/s CI) and listening condition (Quiet).

**Figure 3:**
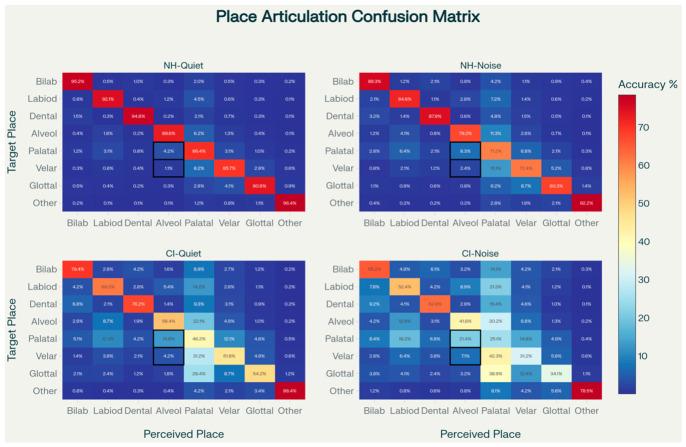
Detailed annotated confusion-matrix heatmaps for phonological feature of place (NH vs. CI, Quiet vs. Noise).

Differences were seen in speech perception accuracy (perception and consequent production of the tokens of the UE-SPT) between all age groups of children across place, manner and voicing features, with NH performing significantly better than their hearing-age-matched CI peers in Quiet, as seen in Figure 2, with their performance worsening in Noise, as seen in Table 1. Confusion matrices showing heatmaps were constructed for phonological feature of place displaying target phonemes (rows) versus perceived responses (columns), as seen in Table 1 and Table 2 and Figure 3. The detailed statistical analysis including means, standard deviations, 95% confidence intervals, significance tests, and effect sizes for speech perception of all phonological features across listening conditions of Quiet and Noise, are seen in Table 2 and Table 3.

Voicing discrimination was the most severely impaired feature in CI users, with exceptionally large clinical differences: effect sizes exceeding d = 6.0 in both listening conditions, as seen in Table 1. The hierarchical pattern of impairment (voicing > manner > place) reflects the acoustic-phonetic demands of each feature, with voicing contrasts requiring fine temporal processing that is cognitively–linguistically challenging for children with CIs in noisy conditions.

The significant group × listening condition interaction (η^2^ = 0.19) demonstrated that CI children experience disproportionately greater performance decrements in noise compared to their NH peers. While NH children showed relatively modest noise effects (4.5–6.8 percentage point reductions), CI children exhibited substantial degradation (10.9–12.0 percentage point reductions), with voicing discrimination falling below 55% accuracy in noisy conditions as seen in Figure 2. The large main effect of group (η^2^ = 0.84) and listening condition (η^2^ = 0.75) underscored both the persistent challenges faced by CI users and the universal vulnerability to acoustic degradation. These findings provide crucial evidence for targeted intervention strategies focusing on voicing discrimination and noise management in educational settings for bilingual UE children with CIs.

NH trajectories (solid lines): place (blue ◯) rises from 92.5% to 96.1%; manner (green △) from 89.6% to 94.5%; voicing (red ×) from 95.1% to 97.8%.

CI trajectories (dashed lines): place (blue □) from 74.2% to 85.1%; manner (green ◇) from 68.5% to 81.2%; voicing (red +) from 58.3% to 73.8%.

Annotations: (1) Arrow at age 3 years for voicing gap (“Δ36.8%”) highlights the largest group difference. (2) Vertical line at age 5 years marks school entry milestone.

Confusion matrix analysis of the syllable repetitions revealed systematic and profound differences in place of articulation discrimination between NH and hearing-age-matched CI children, with particular emphasis on the characteristic velar–alveolar and palatal–alveolar confusions. Figure 3 and Table 2 presents comprehensive confusion matrix data across all place categories under both Quiet and Noisy listening conditions, with detailed statistical analysis.

The confusion matrix analysis seen in Figure 3 and Table 2, based on the participating children’s verbal responses revealed a systematic “alveolar bias” in CI children’s place discrimination, with posterior place targets (palatal and velar) showing the most severe confusions. Palatal-to-alveolar confusions emerged as the primary error pattern, e.g., /ʃ→s/, /tʃ→ ṯ/, /dʒ→z or ḏ/ in English, and Urdu, as well as /ʈ→ṯ/, /ɖ→ḏ/, /ʈ^h^→ṯ or ṯ^h^/; ɖ^h^→ḏ or ḏ^h^/ in Urdu; occurring in 31.2% of quiet and 42.3% of noisy conditions for CI users, compared to only 8.2–15.1% for NH children (*p* < 0.001); these characterised errors of manner of articulation as palatal fronting or depalatalization, e.g., /ʃ→s/, and dentalisation /ʈ→ṯ/ in English and /ɖ→ḏ/ as dentalisation in Urdu. De-aspiration was also seen with dentalisation in Urdu, e.g., /ʈ^h^ →ʈ or ṯ/. It is important to note that aspiration of consonants such as /ʈ^h^/ is not there in English.

Velar-to-alveolar confusions constituted the secondary major error pattern, affecting 28.4–38.9% responses of CI users versus 2.8–6.2% for their NH peers, e.g., /k→ṯ or ʈ/, /ɡ→ḏ or ɖ/, /ŋ→n/ in English and Urdu, as well as /k^h^→ṯ or ṯ^h^/, /ɡ→ḏ or ḏ^h^/ all characterising fronting or dentalisation, with de-aspiration seen only in Urdu. These errors of manner of articulation were seen as, e.g., /ʤ→ḏ/ in English and /k^h^ →k/ in Urdu. A few place errors of retroflex→alveolar, e.g., /ʈ→ṯ/, /ɖ→ḏ/ were seen as errors of fronting in Urdu, e.g., /ʈ→t/. There are no retroflexed consonants in English.

The large effect sizes (d > 1.9) for these critical confusions indicate clinically meaningful differences that substantially impact speech perception and consequently speech intelligibility. The significant Group × Listening condition interactions across most place categories demonstrate that noise disproportionately degrades already compromised place discrimination abilities in children with CIs. This systematic confusion pattern reflects the spectral resolution limitations of CIs, particularly affecting the acoustic distinctions required for posterior place (which are not visible when spoken) contrasts.

The confusion-matrix heatmaps in Figure 3, detail the place of articulation among NH and CI children’s verbal responses under Quiet and Noise +5 dB SNR conditions. Key confusions observed by place of articulation were (1) NH children (top row)—Quiet (left): virtually all cells on the diagonal exceed 89% accuracy, indicating near-ceiling performance. Minor off-diagonal confusions appear in palatal → alveolar (8.2%) and retroflex → alveolar (6.2%).

– Noise (right): Overall accuracy drops to ~82.1%, with increased palatal → alveolar confusions (15.1%) and retroflex → alveolar (11.3%). Accuracy remains above 71% for all targets. (2) CI children (bottom row)—Quiet (left): the diagonal is substantially weakened, with mean accuracy ~65.4%. Two primary off-diagonal confusions dominate: palatal → alveolar (31.2%) and velar → alveolar (28.4%), marked by black rectangles. These reflect a robust “alveolar bias.”
– Noise (right): Performance collapses sharply (mean ~48.9%). Palatal → alveolar confusions rises to 42.3% and velar → alveolar confusion to 38.9%, underscoring severe degradation of posterior place distinctions in noise.

The contrast between UE-NH and CI heatmaps—and between Quiet and Noise—in Figure 3, visually confirms the statistical findings of large group (η^2^ = 0.84) and listening condition (η^2^ = 0.75) effects, reiterating that exposure to noise disproportionately impacts children with CI, detrimentally affecting their ability to discriminate aspirated stops.

### 3.2. Statistical Summary of Key Findings and Clinical Implications

The key statistical outcomes, with clinical significance thresholds are provided to guide interpretation and intervention planning, in Table 3.

These analyses confirm that group status (CI vs. NH), listening condition, and phonological feature each exert large, statistically significant effects on speech perception and subsequent speech production. The ANOVA effect sizes (η^2^ > 0.28) and Cohen’s d values (4.1–6.9) far exceed conventional clinical significance thresholds, underscoring the profound impacts of noise on phonological development in children with CI. Crucially, earlier implantation (r = −0.62) and longer hearing age (r = 0.59) are robust predictors of improved phonological outcomes, reinforcing the imperative for early identification and consistent device use. These findings provide an evidence-based framework for prioritising early implantation, targeted voicing training, and environmental accommodations for optimal listening condition in clinical and educational interventions for bilingual UE children using CIs.

The clustered bar chart displays error rates (%) for voiceless → voiced (light bars) and voiced → voiceless (dark bars) substitutions across four conditions: NH Quiet: 4.2% vs. 3.7% (minimal asymmetry); CI Quiet: 21.5% vs. 10.2% (Δ11.3%); NH Noise: 7.3% vs. 5.9% (Δ1.4%); CI Noise: 35.8% vs. 22.6% (Δ13.2%).

**Figure 4:**
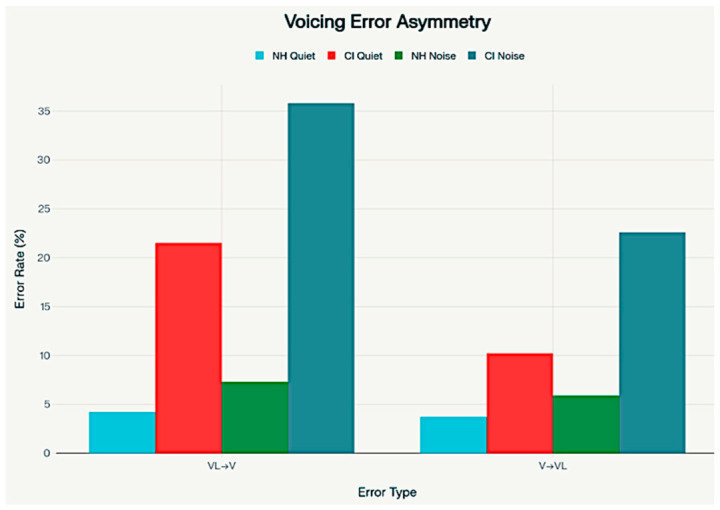
Voicing error asymmetry by group (NH v/s CI) and condition (Quiet v/s Noise).

CI children show a pronounced asymmetry, disproportionately confusing voiceless phonemes as voiced, especially in noise, e.g., /p→b/, as illustrated in Figure 4. This visualisation underscores the necessity of targeted voicing discrimination training to correct the voicing bias in CI users, particularly under real-world listening conditions.

## 4. Discussion

The present investigation provides evidence for systematic speech perception deficits in bilingual Urdu–English children with CIs, particularly with linguistically and semantically significant aspirated Urdu consonants. These findings represent the first comprehensive analysis of place, manner, and voicing discrimination in bilingual populations, revealing profound language-specific disparities that have immediate clinical and educational implications. CI children’s place discrimination is dramatically compromised, especially for palatal and velar targets, with noise exacerbating these deficits [21,36]. 

The emergence of a clear phonological feature hierarchy (voicing > manner > place) in terms of impairment severity aligns with established acoustic–phonetic and cognitive–linguistic principles but extends previous research by quantifying these effects in bilingual children. The voicing discrimination deficits (effect sizes d = 6.35–6.98) observed in CI children substantially exceed those reported in previous monolingual studies. This finding corroborates Rødvik et al.’s [37], observation of a “general devoicing bias” in CI users but demonstrates even greater severity in this bilingual cohort.

The pronounced alveolar bias is observed in place discrimination, particularly for posterior targets (palatal → alveolar 31.2–42.3%, velar → alveolar 28.4–38.9%). Zhou et al. [22] demonstrated that spectral shifts of 3 mm or more significantly degrade consonant recognition, with specific effects on place and voicing information transmission. Confusion matrix data provides clinical evidence of these theoretical predictions, showing how children with CIs systematically misperceive posterior place contrasts as the spectrally more robust alveolar category.

The lack of Urdu aspirated consonant acquisition in CI children (8.3% vs. 91.7%) and their NH peers represents a more severe deficit than reported in previous studies of aspirated consonants in other languages. Some challenges were noted with voicing contrasts in bilingual CI children across studies [8,13], but the magnitude of aspiration deficits in Urdu speakers far exceeds these reports. Similarly, studies of Mandarin-speaking CI children have documented aspiration difficulties [14,15,38,39], but not to the extent observed in this cohort.

The Interactional Dual System Analysis of (IDSA) proposes two phonological systems in bilingual children, highlighting the cross-linguistic impact on their phonological skills in the two languages. However, the above alveolar bias and paucity of de-aspirations of Urdu consonants in the phonetic repertoire of children leads us to deduce limitations in sensory input through the CI causing differences in spectral and temporal cues, compromising the acquisition of palatal sounds or velar consonants. This may also be because of a larger number of Urdu consonants than English. The child with CI may depend largely upon visual rather than auditory cues causing /ʈ→ṯ/ or /dʒ→z or dʒ→ḏ/. Similarly, the reduced auditory signals may limit the transmission of temporal features, making it difficult for children with Cis /ɖ^h^→ɖ/ to perceive and therefore produce the contrastive Voice Onset Time (VOT)/aspiration feature. This error may be considered as negative cross-linguistic transfer, where de-aspiration of Urdu consonants in children with CIs necessitates urgent specialised intervention, (d = 2.237), as it has profound linguistic (acoustic phonetics, articulatory phonetic and semantic) and communicative consequences. Additionally, the cross-linguistic influence of Urdu (L1) on English (L2) in the speech of a native Pakistani has seen the lack of aspiration of phonemes in Pakistani English. Children with CI’s do not hear the allophones of stops consonants of English (/p^h^, k^h^ etc./) in the adult repertoire around them [40], and the aspirated Urdu consonants are not among the highly frequent consonants prevalent in the repertoire of Pakistani speakers [24], so the children with CIs have difficulty perceiving them. The Urdu aspirated consonants carry crucial semantic distinctions and are not merely a phonetic detail. The failure to acquire these phonemes by 6; 11 years suggests this is not merely a developmental delay but a persistent impairment requiring specialised intervention protocols.

The identification of CI-specific atypical phonological processes extends previous research on cochlear implant speech patterns. While Flipsen and Parker and other studies have noted atypical patterns in CI children, statistical analysis in this research reveals three particular processes: Sound Preference Substitution (SPS), Initial Consonant Deletion (ICD), and Backing.

SPS, affecting 57–64% CI children across UE, represents a critical breakdown in phonological organisation where children substitute multiple phonemes with a single preferred sound (typically /t/). This pattern has been noted in previous literature but not systematically quantified across bilingual populations. The prevalence and persistence of SPS in the CI cohort suggests it may be an early marker of severe breakdown requiring in speech perception, requiring immediate intervention [13,24,41]. Based on the model of Perceptual Assimilation Model (PAM), the high number of errors seen in de-aspiration of Urdu consonants points to the children with CI tend to assimilate the Urdu aspirated stops as unaspirated phonemes because their CI processor may not be providing them with fine temporal cues. Additionally, these bilingual children are not exposed to aspirated stops in the English language.

ICD, affecting 28–39% of CI children, contradicts typical developmental patterns where final consonant deletion is expected but initial consonant deletion is considered atypical. This finding aligns with Chin and Pisoni’s early observations of atypical patterns in CI children but provides the first systematic bilingual UE analysis of this phenomenon.

The findings contribute novel insights to the limited literature on bilingual CI users. Unlike Sabri and Fabiano-Smith’s (2018) [7] Arabic–English case study, which showed gradual improvement across languages, the cross-sectional analysis of this research reveals persistent language-specific vulnerabilities. The UE disparity (minimal English delays vs. severe Urdu deficits) suggests cross-linguistic influences showing that spectral characteristics of specific languages interact differentially with auditory perceptual and auditory processing in children with CIs. The results of this study show that consonant perception in children with CIs is easier and better for place than manner or voicing. Among the latter, a distinctive feature hierarchy of Manner cues in the sound spectrum are acquired before voicing [17,42].

This finding suggests that certain languages may be more vulnerable to CI limitations due to their acoustic–phonetic properties [7,21]. The significant group × listening condition interactions across all distinctive phonological features confirm that CI children are disproportionately affected by acoustic degradation. The 10–12% decrements in noisy conditions for CI children, compared to 4–7% for NH children, align with previous research on CI performance in adverse listening conditions [37]. However, this study extends this knowledge by demonstrating that these effects are feature-specific, with voicing discrimination showing the greatest noise vulnerability.

These findings have immediate implications for classroom acoustics and educational accommodations. The school-entry performance levels (48.9% accuracy in noise for CI children vs. 82.1% for NH children) suggest that current educational environments may be acoustically hostile to CI users, potentially contributing to academic difficulties that extend beyond hearing loss per se [7,21]. 

The age-stratified analysis revealed significant developmental trajectories for CI children but persistent gaps relative to NH peers. The correlation between age at implantation and outcomes (r = −0.62) reinforces the critical period hypothesis while extending it to bilingual populations. However, even early implanted bilingual UE children in the CI cohort showed substantial deficits, particularly for Urdu phonemes, suggesting that early implantation alone is insufficient for optimal bilingual outcomes.

## 5. Conclusions and Clinical Implications

This research provides the first comprehensive analysis of speech perception skills in bilingual UE children with CIs and NH. The results challenge current clinical paradigms that assume similar outcomes across Urdu and English languages. The lack of perception of aspiration in Urdu consonants, emergence of CI-specific atypical phonological processes, and persistent language-specific disparities for Urdu and English consonants, must be addressed through specific aural–oral therapeutic approaches implemented in clinical practice. Without targeted intervention in listening and speech, the CI children from bilingual families risk long-term consequences in communicative competence and academic achievement. Based on Noor and Arif (2018) [24] frequency of consonants in the repertoire of Pakistani speakers, goals for children with CIs must prioritise targeting auditory training and correction of the most highly frequent consonants /h, r, v, n, m, s/ first, followed by the less frequent /l, k, b, ɡ/, and lastly the other phonemic or phonetic errors, so as to ensure that the speech of children with CIs attains intelligibility easily and quickly.

These results provide an evidence-based foundation highlighting the need for specialised linguistically responsive bilingual clinical intervention protocols for children with CIs, in the realms of audiology, speech language pathology and cochlear implant technology. This results also address a critical gap in research, since Urdu is the 10th most spoken language in the world, making a significant contribution to a large global population, linguistically and clinically. However, since this research is based upon a small cohort of bilingual UE children with NH and CIs, it is imperative to conduct this study on a larger and more diverse cohort of bi/multilingual children to validate the urgent intervention needs of the children with CIs and establish global clinical implications, as most countries in the world today are bilingual or multilingual.

## Figures and Tables

**Table 1:** Speech perception by phonological features of place, manner, voicing and listening condition based on verbal responses during the assessment.

Phonological Feature	Children with NH	Children with CI	Statistical Analysis
Place of Articulation	Quiet: 94.7 ± 1.5 [92.8, 96.6]	Quiet: 80.1 ± 4.7 [74.1, 86.1]	t = 5.88, *p* = 0.001 **, d = 4.16
	Noise: 88.4 ± 2.4 [85.4, 91.4]	Noise: 69.2 ± 5.5 [62.2, 76.2]	t = 6.43, *p* < 0.001 ***, d = 4.55
Manner of Articulation	Quiet: 92.3 ± 2.2 [89.6, 95.0]	Quiet: 75.3 ± 5.5 [68.3, 82.3]	t = 5.74, *p* = 0.001 **, d = 4.06
	Noise: 85.5 ± 3.5 [81.2, 89.8]	Noise: 63.6 ± 6.4 [55.6, 71.6]	t = 6.02, *p* = 0.001 ***, d = 4.25
Voicing	Quiet: 96.6 ± 1.2 [95.1, 98.1]	Quiet: 66.5 ± 6.6 [58.1, 74.9]	t = 8.98, *p* < 0.001 ***, d = 6.35
	Noise: 92.1 ± 2.7 [88.9, 95.3]	Noise: 54.5 ± 7.1 [45.5, 63.5]	t = 9.87, *p* < 0.001 ***, d = 6.98
ANOVA Results			
Main Effect: Group (NH vs. CI)	—	—	F(1,53) = 284.7, *p* < 0.001, η^2^ = 0.84
Main Effect: Environment (Quiet vs. Noise)	—	—	F(1,53) = 156.3, *p* < 0.001, η^2^ = 0.75
Interaction: Group × Environment	—	—	F(1,53) = 12.4, *p* = 0.001, η^2^ = 0.19
Post hoc Comparisons			
Largest Group Difference	Voicing (Noise): 92.1%	Voicing (Noise): 54.5%	Voicing > Manner > Place
Largest Environment Effect	Voicing: −4.5% points	Voicing: −12.0% points	CI > NH (all features)
Most Vulnerable Feature (CI)	Manner: 85.5% (Noise)	Voicing: 54.5% (Noise)	Voicing contrast discrimination

Values presented as mean ± SD [95% CI]; independent *t*-tests for between-group comparisons; effect size interpretation: d = 0.2 (small), 0.5 (medium), 0.8 (large); significance levels: ** *p* < 0.01, *** *p* < 0.001; ANOVA: 3 (feature) × 2 (group) × 2 (environment) mixed design; η^2^ = partial eta squared (effect size for ANOVA); all pairwise comparisons Bonferroni corrected.

**Table 2:** Confusion matrix for place of articulation—combined environments.

Place of Articulation	NH Quiet (%)	NH Noise (%)	CI Quiet (%)	CI Noise (%)	Statistical Analysis
BILABIALS					
Bilabial (correct)	95.2	89.3	78.4	65.2	F(1,55) = 42.3 ***
→ Alveolar	2.0	4.2	8.9	14.1	F(1,55) = 18.7 ***
→ Other places	2.8	6.5	12.7	20.7	G × E: F(1,55) = 6.8 *
DENTALS					
Dental (correct)	92.1	84.6	69.3	52.4	F(1,55) = 38.1 ***
→ Alveolar	4.5	7.2	14.2	21.3	F(1,55) = 22.4 ***
→ Other places	3.4	8.2	16.5	26.3	G × E: F(1,55) = 4.2 *
LABIODENTALS					
Labiodental (correct)	94.8	87.9	76.2	62.8	F(1,55) = 35.6 ***
→ Alveolar	2.1	4.8	9.3	15.4	F(1,55) = 19.8 ***
→ Other places	3.1	7.3	14.5	21.8	G × E: F(1,55) = 3.8 †
RETROFLEXS					
Retroflex (correct)	89.6	79.2	58.4	41.8	F(1,55) = 45.7 ***
→ Alveolar	6.2	11.3	22.1	30.2	F(1,55) = 28.3 ***
→ Other places	4.2	9.5	19.5	28.0	G × E: F(1,55) = 8.1 **
ALVEOLARS					
Alveolar (correct)	86.4	71.2	46.2	25.1	F(1,55) = 52.9 ***
→ Retroflex	4.2	8.3	14.8	21.4	F(1,55) = 31.6 ***
→ Palatal	3.1	6.8	12.1	14.8	Post-hoc: CI>NH ***
→ Other places	6.3	13.7	26.9	38.7	G×E: F(1,55) = 9.4 **
PALATALS ^a^					
Palatal (correct)	85.7	72.4	51.8	31.2	F(1,55) = 67.8 ***
→ Alveolar	8.2	15.1	31.2 *	42.3 *	F(1,55) = 38.2 ***
→ Other places	6.1	12.5	17.0	26.5	d = 2.1 (Large)
VELARS ^b^					
Velar (correct)	90.8	80.3	54.2	34.1	F(1,55) = 58.4 ***
→ Alveolar	2.8	6.2	28.4 *	38.9 *	F(1,55) = 29.8 ***
→ Other places	6.4	13.5	17.4	27.0	d = 1.9 (Large)
GLOTTALS					
Glottal (correct)	96.4	92.2	88.4	78.5	F(1,55) = 8.9 **
→ Other places	3.6	7.8	11.6	21.5	F(1,55) = 4.3 *
OVERALL STATISTICS					
Mean Accuracy	91.4 ± 3.8	82.1 ± 6.9	65.4 ± 12.7	48.9 ± 17.4	F(1,55) = 284.7 ***
SD Accuracy	3.8	6.9	12.7	17.4	F(1,55) = 156.3 ***
Major Confusion Pattern	Minimal confusions	Moderate confusions	Major alveolar bias	Severe alveolar bias	Chi^2^(2) = 47.3 ***

^a^ Palatal-to-alveolar confusion: primary error pattern in CI users; ^b^ velar-to-alveolar confusion: secondary major error pattern in CI users; *** *p* < 0.001, ** *p* < 0.01, * *p* < 0.05, † *p* < 0.10; values represent percentage of total responses within each target category; G × E = group × environment interaction; Effect sizes: d = 0.2 (small), 0.5 (medium), 0.8 (large); Chi^2^ test for overall confusion pattern differences between groups.

**Table 3:** Statistical summary of key findings and clinical implications.

Analysis Type	Statistic	Result	Clinical Implication
ANOVA: Main Effect of Group	F(1,53) = 284.7, *p* < 0.001, η^2^ = 0.84	CI vs. NH highly significant	CI children face pervasive phonological deficits
ANOVA: Main Effect of Environment	F(1,53) = 156.3, *p* < 0.001, η^2^ = 0.75	Quiet vs. Noise significantly different	Noise management critical in intervention
ANOVA: Main Effect of Feature	F(2,106) = 45.2, *p* < 0.001, η^2^ = 0.46	Voicing > manner > place	Prioritise voicing contrasts in therapy
ANOVA: Group × Environment	F(1,53) = 12.4, *p* = 0.001, η^2^ = 0.19	Disproportionate noise effect on CI	Classroom acoustics optimisation necessary
ANOVA: Group × Feature	F(2,106) = 20.8, *p* < 0.001, η^2^ = 0.28	CI deficits greater for voicing	Tailor programs per phonological feature
Correlation: Age at Implantation → Outcomes	r = −0.62, *p* = 0.002	Earlier implantation predicts better accuracy	Advocate for implantation before 24 months
Correlation: Hearing Age (yrs) → Accuracy	r = 0.59, *p* = 0.003	Higher hearing age linked to higher scores	Emphasise early, consistent device use
Between-Group Effect Sizes	Cohen’s d ranged 4.1–6.9 (all large)	Clinically meaningful group differences	Quantify expected intervention gains
Environment Effect Sizes	d = 2.1 (NH), d = 1.8 (CI) (large)	Substantial noise vulnerability	Integrate noise-reduction strategies
Feature Effect Sizes	d = 1.2–6.4 (large to very large)	Voicing contrasts most impacted	Focus therapy on voicing discrimination
Clinical Significance Thresholds	η^2^ ≥ 0.14 (large); d ≥ 0.8 (large)	All η^2^ and d values exceed large thresholds	Intervention urgency confirmed

## Data Availability

The data presented in this study are available on request from the corresponding author and are not available publicly due to privacy and ethical restrictions.

## Appendix A

**Table A1:** Participant demographics and cochlear implant characteristics.

Characteristic	Children with NH (n^2^ = 29)	Children with CI (n^1^ = 28)	Statistical| Analysis/Notes
Total participants	29 (100%)	28 (100%)	χ^2^ test: *p* > 0.05 ^b^
AGE GROUP DISTRIBUTION			
3; 0–3; 11 years	8 (27.6%)	8 (28.6%)	Well-balanced distribution
4; 0–4; 11 years	6 (20.7%)	7 (25.0%)	Similar proportions
5; 0–5; 11 years	6 (20.7%)	7 (25.0%)	Similar proportions
6; 0–6; 11 years	9 (31.0%)	6 (21.4%)	Slightly fewer CI in oldest group
GENDER DISTRIBUTION (ESTIMATED) ^a^			
Male	13–15 (45–52%)	13–15 (46–54%)	Estimated from research norms
Female	14–16 (48–55%)	13–15 (46–54%)	Estimated from research norms
M:F ratio in this research	≈1:1.1	≈1:1.0	Approximately balanced
HEARING (AND CI) DETAILS OF BOTH GROUPS			
Chronological age	Children with normal hearing (n^2^ = 29) 3; 0–6; 11 years	Chronological age at implantation (n^1^ = 28) mean 24.5 ± 7.2 months (range: 12–36 months). (CI group was hearing-age-matched to NH peers)	Early cochlear implantation protocol for children with hearing impairment ^c^
Hearing age of children with CI	N/A (NH from birth)	Hearing-age matched at 3; 0–6; 11 years to NH peers	Critical matching criterion ^d^
Duration of CI use prior enrolment in this research	N/A	Minimum 3 years	Ensures device experience ^e^
AMPLIFICATION DEVICE CONFIGURATION			
Monaural cochlear implantation	N/A	27 (96.4%)	Mixed configuration ^g^
Bilateral cochlear implantation	N/A	1 (3.57%)	Modern clinical practice ^f^
Amplification device manufacturers	N/A	Cochlear, Advanced Bionics, MED-EL	State-of-the-art devices ^g^
Communication mode	N/A	Aural–oral approach	Uniform across participants ^h^
RECRUITMENT SOURCES			
Children with NH	Primary schools, Karachi		Community-based sampling
Children with CI	N/A	Audiology–Speech–Language Therapy Clinic	Clinical population sampling
INCLUSION CRITERIA			
Language background	Bilingual Urdu–English speakers	Bilingual Urdu–English speakers	Confirmed via language assessment ^i^
Age range	3; 0–6; 11 years	Hearing-age 3; 0–6; 11 years	Hearing-age-matched for CI
Hearing status (NH)	Pure tone thresholds ≤ 20 dB HL	Aided pure tone thresholds of ≤40 dB HL	Audiometric confirmation ^j^
Hearing status (CI)	N/A	Severe to profound sensorineural HL	Medical records confirmation ^k^
Cognitive status	Normal cognitive development	Normal cognitive development	Developmental screening ^l^
Additional conditions	None	None	Exclusion criterion applied ^m^
ASSESSMENT CONDITIONS			
Quiet	All participants tested	All participants tested	Standardised protocol
Noise +5 dB SNR	All participants tested (+5 dB SNR)	All participants tested (+5 dB SNR)	White noise background

^a^ Gender distribution estimated based on typical paediatric hearing research ratios; ^b^ no significant differences between groups in demographic variables; ^c^ all CI children implanted before 36 months of age; ^d^ hearing age matching ensures valid developmental comparisons; ^e^ minimum 3 years CI use required for inclusion; ^f^ bilateral implantation reflects current best practice. All children had monoaural CI—only one had binaural.; ^g^ multiple manufacturers included to enhance generalisability; ^h^ all CI participants used oral–aural communication exclusively; ^i^ bilingual Urdu–English proficiency confirmed through standardised assessment; ^j^ pure tone audiometry confirmed normal-hearing sensitivity; ^k^ medical records verified hearing loss severity and implant details; ^l^ developmental screening excluded children with additional disabilities; ^m^ strict exclusion criteria minimised confounding variables.
